# Metabolically Abnormal Non-Obese Phenotype Is Significantly Associated with All-Cause Mortality in Hemodialysis Patients

**DOI:** 10.3390/jcm13041059

**Published:** 2024-02-13

**Authors:** Jin Hyeog Lee, Hae-Ryong Yun, Hyung Woo Kim, Jung Tak Park, Seung Hyeok Han, Yong-Lim Kim, Yon Su Kim, Chul Woo Yang, Nam-Ho Kim, Shin-Wook Kang, Tae-Hyun Yoo

**Affiliations:** 1 Department of Internal Medicine, Yongin Severance Hospital, Yonsei University College of Medicine, Seoul 16995, Republic of Korea; jayhlee@yuhs.ac (J.H.L.); siberian82@yuhs.ac (H.-R.Y.); 2 Department of Internal Medicine, College of Medicine, Institute of Kidney Disease Research, Yonsei University, Seoul 03722, Republic of Korea; drhwint@yuhs.ac (H.W.K.); jtpark@yuhs.ac (J.T.P.); hansh@yuhs.ac (S.H.H.); kswkidney@yuhs.ac (S.-W.K.); 3 Department of Internal Medicine, Kyungpook National University School of Medicine, Daegu 41944, Republic of Korea; ylkim@knu.ac.kr; 4 Department of Internal Medicine, Seoul National University College of Medicine, Seoul 03080, Republic of Korea; yonsukim@snu.ac.kr; 5 Department of Internal Medicine, Catholic University of Korea College of Medicine, Seoul 06591, Republic of Korea; yangch@catholic.ac.kr; 6 Department of Internal Medicine, Chonnam National University Medical School, Gwangju 61469, Republic of Korea; nhk111@chonnam.ac.kr

**Keywords:** kidney failure with renal replacement therapy, metabolic abnormality, obesity, all-cause mortality

## Abstract

The association between obesity and all-cause mortality in patients undergoing kidney failure with replacement therapy (KFRT) has shown conflicting results. This study aimed to evaluate whether metabolic abnormalities (MA) increase the risk of all-cause mortality in these patients. Between 2009 and 2015, 1141 patients undergoing KFRT were recruited from the Clinical Research Center for End-Stage Renal Disease dataset. Patients were divided into four groups according to the presence of obesity and MA. Multivariate Cox proportional hazard analysis was performed to determine the association between the phenotypes and all-cause mortality. During a mean follow-up of 4.2 years, all-cause mortality was observed in 491 (43.0%) patients. Obesity had a 24% decreased risk of all-cause mortality compared with non-obesity. In contrast, the presence of MA showed a 1.53-fold increased risk of all-cause mortality. There was a significant interaction between obesity and MA (*p* = 0.006). In Cox proportional hazard analyses after adjustment of confounding factors, the metabolically abnormal non-obesity (MANO) phenotype showed a 1.63-fold increased risk of all-cause mortality compared with the metabolically healthy non-obesity phenotype. In subgroup analysis, the risk of all-cause mortality was higher in the MANO phenotype; this phenotype was significantly associated with a higher all-cause mortality in patients undergoing KFRT.

## 1. Introduction

The prevalence of obesity has increased considerably, and it is a major clinical and public health concern in many countries. Approximately 20% of the entire adult population of the world will be obese by 2030 [1]. Obesity increases glucose levels and worsens lipid profiles, blood pressure, and the incidence of inflammation [2,3,4]. Consequently, this condition is associated with the worsening of almost all cardiovascular risk factors such as hypertension, type 2 diabetes (T2DM), metabolic syndrome, dyslipidemia, and chronic kidney disease (CKD). Thus, obese individuals have a higher risk of cardiovascular disease (CVD) and all-cause mortality than the general population [5,6].

In contrast, several reports in the past three decades have demonstrated a subtype of obesity without metabolic abnormality (MA), called metabolically healthy obesity (MHO) [7,8,9]. Characteristics of the MHO phenotype include low insulin resistance, low prevalence of dyslipidemia, and a favorable inflammatory profile; despite the adverse effects of obesity on CVD and all-cause mortality, patients with this phenotype often have a better prognosis than those without obesity who have similar clinical conditions [10,11,12]. This association is referred to as “reverse epidemiology” or “obesity paradox” [13,14]. Interestingly, this puzzling association is also observed in patients undergoing kidney failure with replacement therapy (KFRT) [14].

These results led us to assume that all-cause mortality differed according to the presence of obesity and metabolic disturbances in KFRT patients. Therefore, we aimed to evaluate the complex associations between obesity, metabolic abnormalities, and all-cause mortality in these patients. In addition, we evaluated whether the association between obesity and all-cause mortality could be modified by the presence of MA.

## 2. Materials and Methods

### 2.1. Study Population and Design

**Figure 1:**
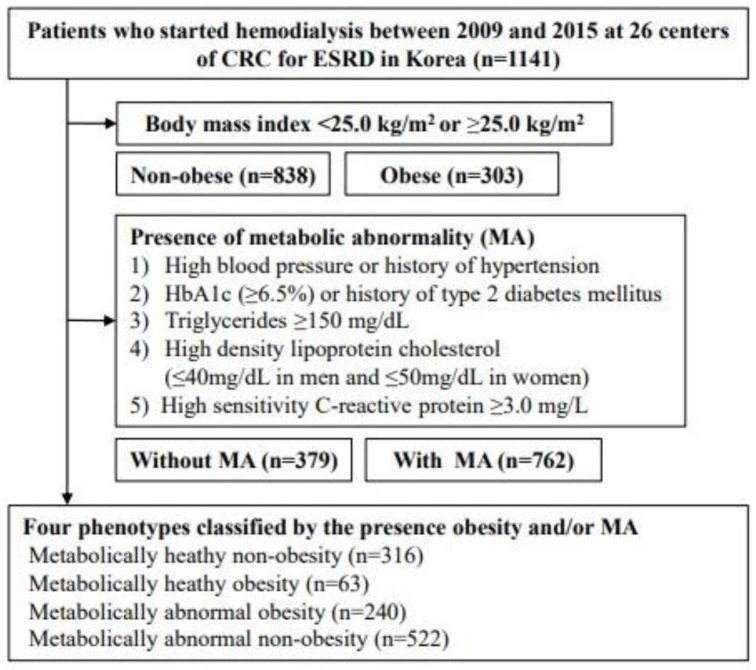
Flow diagram of study patients. Abbreviations: CRC for ESRD, Clinical Research Center for End Stage Renal Disease.

The Clinical Research Center (CRC) for End-Stage Renal Disease (ESRD) conducted a multicenter prospective observational cohort study in South Korea. Clinical and laboratory data were collected from 26 centers. Patients who commenced dialysis between 2009 and 2015 were recruited from the CRC registry for the ESRD dataset. A total of 2205 patients were enrolled in this study. All enrolled patients were adults aged > 20 years who had commenced renal replacement therapy for kidney failure; 1064 patients were excluded if they were scheduled to undergo kidney transplantation within three months or if they had missing covariate parameters. Finally, 1141 patients undergoing incident hemodialysis were included in this study (Figure 1).

All patients provided written informed consent to voluntarily participate in this study, and all investigations were conducted in accordance with the guidelines of the 2008 Declaration of Helsinki. This study was approved by the institutional review boards of the participating centers.

### 2.2. Data Collection

Demographic and laboratory data were collected at enrollment and at every 6 or 12 months thereafter. Demographic data included age, sex, height, body weight, body mass index (BMI), smoking status, medical history, Charlson comorbidity index, systolic blood pressure (SBP), and diastolic blood pressure (DBP) at the time of study entry. The BMI was calculated by dividing the initial body weight (kg) by the squared body height (m^2^). Smoking history was defined as currently or formerly smoking. To collect demographic data, chart reviews and informal interviews were conducted by data coordinators who had been trained in the participating centers. Pre-dialysis venous blood samples were collected from patients in a clinically stable state. Blood samples were obtained to measure hemoglobin, glucose, hemoglobin A1c (HbA1c), albumin, calcium, phosphate, uric acid, total cholesterol, triglycerides, high-density lipoprotein cholesterol (HDL-C), intact parathyroid hormone (PTH), and high-sensitivity C-reactive protein (hs-CRP) levels.

### 2.3. Definitions of Obesity and Metabolic Abnormality

Obesity was defined as a BMI > 25.0 kg/m² as recommended by the Steering Committee of the Regional Office for the Western Pacific Region of the World Health Organization. In 2000, the International Association for the Study of Obesity and International Obesity Task Force proposed the appropriateness of classifying obesity in Asia [15].

There are no universal criteria for defining MA. High blood pressure or a history of hypertension; lipid profiles including HDL-C, LDL-C, and/or triglycerides; insulin resistance defined by fasting glucose; homeostatic model assessment of insulin resistance; HbA1c; presence of type 2 diabetes mellitus; and inflammatory markers have been used to define MA in previous studies [10,12,16,17,18]. In addition, most studies adopted an abnormal cut-off value for each measure as recommended by the Adult Treatment Panel-III [19]. In this study, MA was defined as the presence of three or more of the following factors: (1) high blood pressure (≥140/80 mmHg), previous history of hypertension, or use of antihypertensive medication; (2) fasting plasma glucose concentration of ≥126 mg/dL, HbA1c ≥6.5%, or previous history of T2DM; (3) triglyceride ≥ 150 mg/d or use of lipid-lowering drugs; (4) HDL-C ≤ 40 mg/dL in men or ≤50 mg/dL in women; and (5) hs-CRP ≥ 3 mg/L. Based on this definition, patients were divided into four phenotypes: metabolically healthy non-obesity (MHNO), MHO, metabolically abnormal obesity (MAO), and metabolically abnormal non-obesity (MANO).

### 2.4. Primary Outcome of Interest

The primary outcome of this study was all-cause mortality up to 31 December 2019. All-cause mortality was ascertained from records linked to the Korean Statistical Information Service using unique personal identification numbers. The participants were dropped at death, when they underwent kidney transplantation, or when lost to follow-up, whichever occurred first.

### 2.5. Statistical Analysis

Continuous variables are expressed as mean ± standard deviation or median ± interquartile range. Categorical variables are expressed as the number of participants and percentages. To compare the differences among the presence of obesity, metabolic disturbances, and the four phenotypes, one-way analysis of variance and the chi-square test were used for continuous and categorical variables, respectively. The probability of survival rate was compared using a Kaplan–Meier curve. Survival time was defined as the interval between enrollment and first onset of all-cause mortality. Multivariate Cox proportional hazards regression models were constructed to assess the risk of all-cause mortality. Model 1 represents the crude risk without adjustment. Model 2 was adjusted for age, sex, and smoking status. Model 3 was further adjusted for SBP, DBP, hemoglobin, albumin, total cholesterol, phosphate, uric acid, intact-PTH, and hs-CRP levels, in addition to model 2. The results of the multivariate Cox proportional hazards regression models are presented as hazard ratios (HRs) and 95% confidence intervals (CIs). Violation of the proportional hazard assumption was tested by evaluating log (–log [survival]) curves. Patients lost to follow-up were censored on the day of the final examination. We further tested the association between each phenotype and all-cause mortality among prespecified subgroups by sex (female or male) and age (<60 or ≥60 years). Statistical analyses were performed using Stata, version 15.1 (Stata Corporation, College Station, TX, USA). Statistical significance was set at *p* < 0.05.

## 3. Results

### 3.1. Baseline Characteristics

The demographic and clinical characteristics of patients in the four phenotype-based groups are shown in Table 1.

The mean age of the study participants was 56.5 ± 13.9 years, and 705 (61.8%) patients were men. The prevalence of MHNO, MHO, MAO, and MANO phenotypes was 316 (27.6%), 63 (5.5%), 240 (21.0%), and 522 (45.7%), respectively. Patients with MANO were significantly older, had a higher incidence of T2DM (*p* < 0.001), and had higher MA parameters than those with other phenotypes. However, history of hypertension and blood pressure did not differ significantly among the phenotypes.

### 3.2. Obesity, Metabolic Abnormality, and All-Cause Mortality

First, we evaluated the association between obesity and all-cause mortality. During follow-up, all-cause mortality was observed in 378 (45.1%) and 113 (37.2%) patients without and with obesity, respectively. Unadjusted HRs were significantly associated with a decreased risk of all-cause mortality in patients with obesity compared with those without obesity. These associations were consistent after adjusting for confounding factors (HR, 0.76; 95% CI, 0.59–0.97; *p* = 0.03; Table 2).

We further analyzed the association between MA and all-cause mortality. Among patients without MA, 112 (29.5%) reached the study endpoint; in contrast, 379 (49.7%) patients with MA reached the study endpoint. The multivariate Cox model showed that MA was significantly associated with an increased risk of all-cause mortality (HRs, 1.53; 95% CI, 1.20–1.96; *p* = 0.001). Interestingly, significant interactions between obesity and MA were observed when the interaction effects between these two factors were analyzed using a multivariate Cox model (*p* = 0.006).

### 3.3. Risk of All-Cause Mortality According to the Presence of Obesity and Metabolic Abnormality

Finally, we investigated whether the association between obesity and all-cause mortality could be modified by MA. Within 4749.2 patient-years, 491 (43.0%) all-cause mortalities occurred. All-cause mortality was observed in 93 (29.4%), 19 (30.1%), 94 (39.1%), and 285 (54.5%) patients in the MHNO, MHO, MAO, and MANO groups, respectively (*p* < 0.001). In a crude model, the MAO and MANO groups were associated with a 1.45-fold (95% CI, 1.09–1.93; *p* = 0.01) and 2.17-fold (95% CI, 1.71–2.74; *p* < 0.001) increase, respectively, in the risk of all-cause mortality compared with the MHNO group (Model 1). In multivariate Cox proportional analyses after adjusting for confounding factors, MANO was associated with a 1.63-fold (95% CI, 1.25–2.13; *p* < 0.001) increase in the risk of all-cause mortality compared with the MHNO group (Table 3).

**Figure 2:**
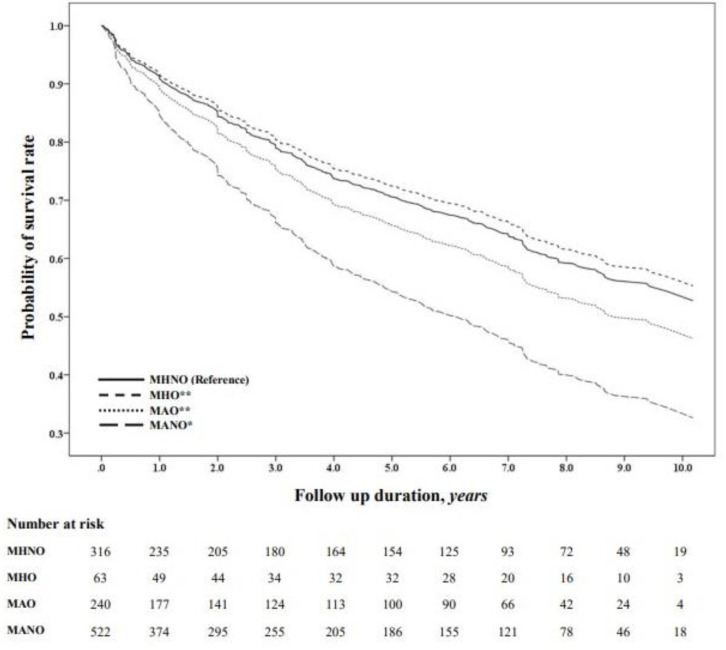
Kaplan–Meier survival probability across the metabolic phenotypes. The probability of survival was compared using Kaplan–Meier curves. * *p* < 0.001 versus reference; ** *p* = 0.001 versus MANO. Abbreviations: MHNO, metabolically healthy non-obesity; MHO, metabolically healthy obesity; MAO, metabolically abnormal obesity; MANO, metabolically abnormal non-obesity.

In contrast, the MHO and MAO groups did not show statistically significant differences compared with the MHNO group. The probability of survival was also significantly lower in the MANO group than in the MAO (*p* = 0.001), MHO (*p* = 0.001), and MHNO (*p* < 0.001) groups (Figure 2).

### 3.4. Subgroup Analyses

**Figure 3:**
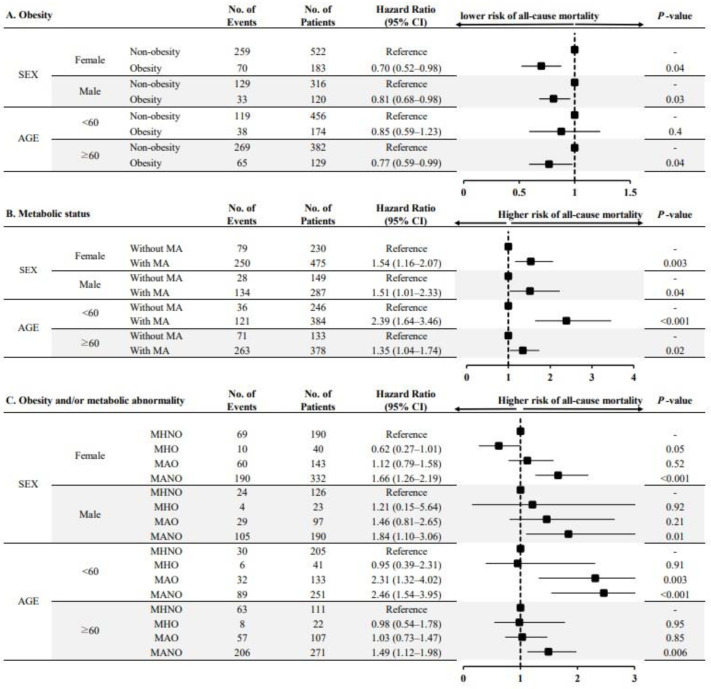
Multivariable-adjusted hazard ratios for all-cause mortality according to obesity and/or metabolic status, stratified by subgroups. Note: (**A**) HRs for all-cause mortality according to the presence of obesity; (**B**) HRs for all-cause mortality according to the presence of metabolic abnormalities; (**C**) HRs for all-cause mortality among the four phenotypes classified according to the presence of obesity and/or metabolic abnormality. Each HR was adjusted for confounding factors except for the corresponding subgroups. Abbreviations: No, number; CI, confidence interval; MA, metabolic abnormality; MHNO, metabolically healthy non-obesity; MHO, metabolically healthy obesity; MAO, metabolically abnormal obesity; MANO, metabolically abnormal non-obesity.

To test the robustness of our primary results, a subgroup analysis was performed based on the prespecified subgroups. The risk of all-cause mortality was significantly lower in obese patients (both female and male patients) than in non-obese patients (Figure 3A). The presence of MA was associated with an increased risk of all-cause mortality irrespective of sex (Figure 3B). This association was consistently observed in younger and older patients receiving KFRT. When we stratified the four phenotypes according to the presence of obesity and MA, the MANO group was associated with an increased risk of all-cause mortality compared with the MHNO group in most of the prespecified subgroups (Figure 3C).

## 4. Discussion

This study examined the association between obesity and/or metabolic unhealthiness and all-cause mortality in patients who underwent KFRT. Obesity was significantly associated with decreased risk of all-cause mortality. By contrast, MA was significantly associated with an increased risk for adverse clinical outcomes. Interestingly, an interaction effect between obesity and MA was observed. In addition, the risk of all-cause mortality was particularly evident in non-obese patients with metabolic disturbances. These associations were consistent in most prespecified subgroups. These findings suggest that lower BMI and metabolic disturbances synergistically affect all-cause mortality in patients undergoing KFRT.

In the general population, obesity and being overweight are associated with a higher risk of all-cause mortality. Obesity induces insulin resistance and inflammation, which increase the risk of cardiovascular disease and death in the general population [20]. In a systematic review and meta-analysis of studies involving approximately 10.6 million individuals, the associations of overweight and obesity with higher mortality rates were consistent across four continents [21]. This association was also observed in patients who did not receive KFRT. In the analysis of 453,946 United States veterans with eGFR < 60 mL/min per 1.73 m^2^, BMI showed a U-shaped association with CKD progression and all-cause mortality. Thus, lower BMI and extreme obesity are associated with an increased risk of adverse clinical outcomes [22]. Similarly, the risk of mortality was higher in obese patients with KFRT, irrespective of the dialysis modality [23,24,25]. However, the clinical effects of a higher BMI have not been consistently observed in these patients. Fleischmann et al. first reported that KFRT patients with a higher BMI had a higher survival rate than those with a normal BMI [26]. Park et al. demonstrated that mortality risk was lower with a higher BMI among 20,818 patients on long-term dialysis in South Korea [27]. In addition, Kim et al. also reported that time-varying obese hemodialysis patients had better survival during the early post-dialysis period [28]. Such contradictory findings regarding obesity and mortality can be partly explained by differences in the study population. However, discrepancies in unmeasured aberrant metabolic components between study cohorts may have affected the differences between obesity and adverse clinical outcomes. Patients undergoing KFRT have many cardiovascular risk factors, such as hypertension, T2DM, and metabolic syndrome. Therefore, it is not surprising that these patients have a higher risk of CVD and a higher mortality rate than the general population. Therefore, we focused on different types of obesity and investigated whether clinical outcomes differed depending on the presence of MA. In this study, the patients with obesity had better clinical outcomes than those without obesity. Additionally, patients without MA had better outcomes than those without MA. The significant interaction between obesity and MA suggests that MA negatively affects BMI maintenance, resulting in an increased risk of adverse clinical outcomes in the dialysis population. Therefore, our results clearly showed that the risk of all-cause mortality was highest in dialysis patients without obesity who had MA.

However, the protective role of obesity in patients undergoing KFRT remains unclear. This puzzling association has also been reported in chronic diseases such as heart failure and chronic obstructive pulmonary disease [29,30]. Hence, there must be prevailing conditions that are uniquely present not only in patients with KFRT but also in similar populations. Additionally, the obesity paradox may indicate that other factors underlie the traditional association between obesity and adverse clinical outcomes. One possible explanation for this is protein-energy wasting (PEW). The International Society of Renal Nutrition and Metabolism recommends this term because, unlike malnutrition, PEW cannot be corrected with nutritional support and is associated with the continuous loss of muscle and fat mass, which are useful fuel reserves [31]. In addition, inflammation promotes PEW in patients undergoing KFRT [32,33,34]. Sustained release of inflammatory cytokines such as interleukin-6 or tumor necrosis factor-α may cause muscle and fat mass wasting and hypoalbuminemia in patients with metabolic disturbances [33]. Thus, an underweight status may potentially reflect the magnitude of PEW and/or inflammation in dialysis patients. In this study, patients with MANO had lower serum albumin and higher hs-CRP levels than those in the other groups. These findings provide evidence of preexisting PEW in this group.

Our study had several limitations. First, the proportion of patients with MA in this study might have been affected by the definition [35]. In this study, 762 (66.7%) patients had MA; this proportion was higher than that in previous studies involving the general population [36]. However, the prevalence of MA was similar to that reported in our previous study involving patients with CKD who did not undergo KFRT [37]. In addition, we have used four representative metabolic parameters to define metabolic disturbances in several studies and guidelines [38,39]. Second, because this study was a retrospective observational study and not a treatment trial, the causal relationship between metabolic phenotypes and all-cause mortality could not be fully established, and residual confounders remain despite efforts to control important confounders. Third, the BMI could reflect muscle mass, fat mass, and body fluid status; BMI might not provide detailed information about obesity and not be an ideal marker of obesity in patients undergoing dialysis. However, BMI is still widely used as a surrogate marker of obesity in patients undergoing KFRT. Furthermore, other anthropometric markers such as waist-to-hip ratio, waist circumference, and visceral fat thickness could not be verified in our cohort. Finally, we did not assess the contributions of physical activity and fitness to survival. In addition, several lifestyle markers such as dietary composition were not included in our cohort. Further studies are required to address these issues.

## 5. Conclusions

In conclusion, we showed that obesity was associated with favorable clinical outcomes in patients undergoing KFRT. In contrast, MA was associated with an increased all-cause mortality in these patients. Furthermore, all-cause mortality was significantly higher in non-obese patients with MA. Thus, simultaneous stratification according to metabolic abnormalities and obesity may be a useful tool for predicting adverse outcomes in patients undergoing KFRT.

## Figures and Tables

**Table 1:** Baseline characteristics of study patients in four phenotypes classified by the presence obesity and/or metabolic status.

	Overall	MHNO	MHO	MAO	MANO	*p*-Value
Participants, n (%)	1141	316 (27.6)	63 (5.5)	240 (21.0)	522 (45.7)	-
Age (year)	56.5 ± 13.9	53.2 ± 15.3	54.0 ± 14.7	56.2 ± 12.6	58.9 ± 12.9	<0.001
Men, n (%)	436 (38.2)	126 (39.9)	23 (36.6)	97 (40.5)	190 (36.4)	0.64
Smoking habits, n (%)	554 (48.5)	139 (44.6)	31 (49.2)	113 (47.1)	268 (51.7)	0.27
Body mass index (kg/m²)	22.9 ± 3.4	21.1 ± 2.1	26.7 ± 1.7	27.5 ± 2.4	21.6 ± 2.0	<0.001
Hypertension, n (%)	1138 (99.7)	314 (99.3)	63 (100)	240 (100)	521 (99.8)	0.91
Type 2 diabetes mellitus, n (%)	700 (61.3)	73 (10.4)	10 (1.4)	195 (27.8)	422 (60.2)	<0.001
Coronary artery disease, n (%)	165 (14.4)	35 (21.2)	7 (4.2)	33 (20.0)	90 (54.5)	0.03
Congestive heart failure, n (%)	156 (13.6)	36 (23.0)	2 (1.2)	31 (19.8)	87 (55.7)	0.01
Cerebrovascular accident, n (%)	27 (2.3)	4 (14.8)	1 (3.7)	4 (14.8)	18 (66.6)	0.06
Charlson Comorbidity Index	5.3 ± 2.3	4.2 ± 2.2	4.1 ± 2.1	5.5 ± 2.1	6.1 ± 2.2	<0.001
Systolic BP (mmHg)	143.0 ± 23.1	142.8 ± 24.0	140.3 ± 19.5	144.9 ± 22.5	142.6 ± 23.3	0.33
Diastolic BP (mmHg)	77.7 ± 14.3	79.4 ± 15.2	80.4 ± 12.6	79.5 ± 13.1	75.6 ± 14.7	<0.001
Hemoglobin (g/dL)	8.7 ± 1.6	8.6 ± 1.7	8.9 ± 1.8	8.8 ± 1.4	8.7 ± 1.6	0.30
Calcium (mg/dL)	7.7 ± 1.0	7.6 ± 1.1	7.4 ± 1.2	7.7 ± 1.0	7.7 ± 1.0	0.03
Phosphate (mg/dL)	5.6 ± 1.9	5.7 ± 2.0	6.1 ± 1.9	5.9 ± 2.0	5.4 ± 1.8	0.003
Albumin (g/dL)	3.3 ± 0.3	3.4 ± 0.6	3.5 ± 0.5	3.3 ± 0.03	3.1 ± 0.5	<0.001
Uric acid (mg/dL)	8.2 ± 2.6	8.2 ± 2.6	8.4 ± 2.6	8.7 ± 2.4	8.1 ± 2.6	0.03
HbA1c (%)	6.0 ± 1.4	5.3 ± 0.8	5.1 ± 1.0	6.2 ± 1.4	6.4 ± 1.5	<0.001
Total cholesterol (mg/dL)	156.8 ± 48.7	157.9 ± 50.0	150.2 ± 38.5	157.1 ± 53.5	156.2 ± 44.6	0.69
Triglyceride (mg/dL)	126.7 ± 75.5	92.5 ± 42.5	97.9 ± 37.6	150.9 ± 83.6	139.6 ± 82.2	<0.001
HDL cholesterol (mg/dL)	40.0 ± 14.2	48.7 ± 14.3	43.6 ± 20.3	33.8 ± 10.0	37.2 ± 12.3	<0.001
hs-CRP (mg/L)	0.4 (0.1−1.9)	0.26 (0.06−0.8)	0.29 (0.09−1.1)	0.46 (0.1−2.3)	0.61 (0.1−3.5)	<0.001
Intact-PTH (pg/mL)	199.6 (108.2−329.5)	244.8 (132.8−373.9)	307.0 (147.0−483.7)	211.9 (110.2−352.4)	166.5 (91.3−280.6)	<0.001

Continuous variables are expressed as mean ± SD or median (interquartile range). Categorical variables are expressed as the number of participants and percentages. Smoking was defined as never smoking, currently smoking, or formerly smoking. Abbreviations: MHNO, metabolically healthy non-obesity; MHO, metabolically healthy obesity; MAO, metabolically abnormal obesity; MANO, metabolically abnormal non-obesity; HbA1c, hemoglobin A1c; HDL, high-density lipo-protein; hs-CRP, high-sensitivity C-reactive protein; PTH, parathyroid hormone.

**Table 2:** Hazard ratios for all-cause mortality according to the presence of obesity and metabolic status.

		BMI Category		Metabolic Status	
		Non-Obesity	Obesity	Without MA	With MA
Patient-years		3448.3	1300.9	1758.2	2990.9
Incidence of outcome, n/n		378/838	113/303	112/379	379/762
Incidence rate per 100 patients-years		10.9	8.6	5.2	11.4
Model 1	HRs (95% CI)	1.00 (Reference)	0.71 (0.57–0.90)	1.00 (Reference)	1.93 (1.56–2.38)
	*p*-value	-	0.002	-	<0.001
Model 2	HRs (95% CI)	1.00 (Reference)	0.77 (0.61–0.96)	1.00 (Reference)	1.60 (1.29–1.96)
	*p*-value	-	0.02	-	<0.001
Model 3	HRs (95% CI)	1.00 (Reference)	0.76 (0.59–0.97)	1.00 (Reference)	1.53 (1.20–1.96)
	*p*-value	-	0.03	-	0.001

Model 1: a crude analysis without adjustment. Model 2: adjusted for age (≥60 years or <60 years), sex, and smoking status. Model 3: adjusted for model 2 plus blood pressure, hemoglobin, glucose, albumin, phosphate, uric acid, total cholesterol, intact PTH, and hs-CRP. Abbreviations: BMI, body mass index; MA, metabolically abnormal; HRs, hazard ratios; CI, confidence interval; PTH, parathyroid hormone; hs-CRP, high-sensitivity C-reactive protein.

**Table 3:** Hazard ratios for all-cause mortality among four metabolic subtypes classified by the presence of obesity and/or metabolic abnormality.

		MHNO	MHO	MAO	MANO
Patient-years	4749.2	1457.8	300.4	1000.5	1990.4
Incidence of outcome, n/n	491/1141	93/316	19/63	94/240	285/522
Incidence rate per 100 patient-years	10.3	6.3	6.3	9.3	14.3
Model 1	HRs (95% CI)	1.00 (Reference)	0.99 (0.61–1.63)	1.45 (1.09–1.93)	2.17 (1.71–2.74)
	*p*-value	-	0.99	0.01	<0.001
Model 2	HRs (95% CI)	1.00 (Reference)	0.92 (0.56–1.52)	1.26 (0.95–1.68)	1.71 (1.35–2.16)
	*p*-value	-	0.75	0.11	<0.001
Model 3	HRs (95% CI)	1.00 (Reference)	1.02 (0.62–1.69)	1.30 (0.95–1.80)	1.63 (1.25–2.13)
	*p*-value	-	0.92	0.10	<0.001

Model 1: a crude analysis without adjustment. Model 2: adjusted for age (≥60 years or <60 years), sex, and smoking status. Model 3: adjusted for model 2 plus blood pressure, hemoglobin, glucose, albumin, phosphate, uric acid, total cholesterol, intact PTH, and hs-CR. Abbreviations: MHNO, metabolically healthy nonobesity; MHO, metabolically healthy obesity; MAO, metabolically abnormal obesity; MANO, metabolically abnormal non-obesity; HRs, hazard ratios; CI, confidence interval; PTH, parathyroid hormone; hs-CRP, high-sensitivity C-reactive protein.

## Data Availability

Due to the ethical restrictions imposed by the Institutional Review Board of the Kyungpook National University Hospital, data are available upon request.
